# Valuing patients’ high unmet need to drive innovative therapies for inherited retinal degenerations: insights from the RIWC 2024 workshop on value

**DOI:** 10.3389/fmed.2025.1481670

**Published:** 2025-03-04

**Authors:** Nabin Paudel, Avril Daly

**Affiliations:** Retina International, Dublin, Ireland

**Keywords:** Inherited Retinal Degeneration (IRD), value, innovation, patient voice, regulation and health technology assessment

## Abstract

The importance of patient involvement in the therapeutic development ecosystem is being increasingly recognized, however not all stakeholders are fully aware of the unique perspective that patients can bring to these platforms. In this perspective article that is based on a workshop organized at the Retina International World Congress (RIWC) in Dublin in June 2024, we discuss the interpretation of value from patient’s perspective, challenges in the development of innovative medicines such as cell and gene therapies for Inherited Retinal Degenerations, the resources required to bring a drug to market, and the need to incorporate patient voice throughout the drug development pathway from pre-clinical studies to clinical trial designs, regulatory and health technology assessments decisions. We hope that this article will increase awareness among all relevant stakeholders including patients, clinicians, scientists, developers, regulators, decision makers and industry representatives on the importance of involving patients in the developmental lifecycle of novel therapies so that therapies are developed that make a meaningful improvement in patients’ lives.

## Introduction

Incorporating the patient voice in the biomedical innovation ecosystem is critical to improving outcomes and reducing research waste (1). The benefit of valuing the patient’s perspective in clinical trial design, regulatory and health technology assessment (HTA) decisions is well recognized (2). However, not all patients are informed about the value they can bring to improving the innovation process and how their experience, expertise and the impact of their condition at various phases of their journey can improve the understanding of their needs and lead to improvement in their outcomes. Clinical experts, researchers and developers, including industry representatives, recognize the need to be better informed about the importance of potential patients and public involvement in research and development (3).

This perspective article is based on the output from a half day workshop conducted at the Retina International World Congress (RIWC2024), June 5–8, 2024, in Dublin, Ireland (4). The purpose of the workshop was to raise awareness among the clinical, industry and patient leaders about the crucial role patients can play in healthcare decision making and the need to change the traditional perception of impact and value of potential therapeutic solutions targeting inherited retinal degenerations. Inherited retinal degenerations (IRDs) are a group of diseases that affect the specialized cells in the retina that are responsible for the early steps of vision. Most of these diseases progress slowly over many years which leads to challenges in determining the benefit of an intervention within a short time frame as well as determining value for high-cost treatments such as cell and gene therapies.

The workshop was attended by a combination of 55 patient leaders, leading clinical experts and industry partners working in the field of inherited retinal degenerations from across the globe.

## Value – the patient’s perspective

The session started off with a definition of value in health care decision making which has been interpreted differently by different stakeholders. For regulators, payers, industry leaders and decision makers, value means reducing costs to the healthcare systems or generating profits (5). For patients with retinal degenerations, particularly those living with Inherited Retinal Degenerations (IRDs) that progress slowly over time, the traditional definition of value is not appropriate, as it is based on interaction with the healthcare system. Patients living with IRDs may not regularly visit eye clinics as they are often told that ‘nothing can be done.’ This leads to a perception of IRDs being ‘low cost’ to the healthcare system whereas in fact people living with IRDs more frequently avail of social care and services that are accounted for separately in many countries. In utilizing this method of assessment, therapeutic interventions for IRDs will always be considered as low value which in itself creates a barrier to access for many patients.

For people living with IRDs, value needs to incorporate the broader impact of these conditions on quality of life, employment as well as emotional well-being in patients’ lives as well as that of their families (6). Previous studies undertaken by Retina International report that most of the economic burden on IRDs is from the impact on well-being and productivity rather than the cost of healthcare delivery (7, 8). Incorporating broader elements such as quality of life and societal impact of a disease in decision making can better reflect the multidimensional value of health interventions to patients and society. Previous reports have suggested implementing modified cost effectiveness analysis models to incorporate the broad societal and equity values in Health Technology Assessments. Some methods include augmented cost-effectiveness analysis (ACEA) and multi-criteria decision analysis (MCDA) to include societal impacts, health equity, emotional well-being, and patient preferences (9).

## Challenges in clinical trial development

Participants expressed the need for the patients’ voice to be included in clinical trial designs and regulatory decision-makings. This involvement ensures that the trials measure the outcomes that are of most value to patients, thereby making treatments more relevant and more effective for the patient. Involving the patient also ensures that regulatory decisions are not based solely on clinical efficacy and safety but also on the patient’s perceived benefit of treatments, achieved through using appropriate patient reported outcome measures. Clinical experts expressed the challenges and frustrations encountered with existing regulatory systems noting that current systems are not tailored to the needs of patients with IRDs but are based on traditional universal standards for all ophthalmic disorders. This issue has been raised in several multistakeholder workshops and meetings over the years (10–12) with no advancement in developing more specific regulatory frameworks for IRDs. There is growing recognition of the importance of patient-reported outcome measures and real-world measures as primary or secondary endpoints in clinical trials, particularly in oncology (13). In ophthalmology, a notable example is the multi-luminance mobility test (MLMT), a real-world mobility test developed based on patient input and used as a primary endpoint in a Phase III gene therapy trial for RPE65 mutation-associated Inherited Retinal Degeneration (14, 15). The MLMT assesses a patient’s navigation and mobility skills across varying light levels, addressing one of the most significant challenges faced by IRD patients. Further innovative outcome measures that accurately reflect patients’ real-world activities and are tailored to specific disease types are urgently needed in the Inherited Retinal Degenerations space. This can only happen via collaborative approaches that actively engage patients in the design and validation of these outcome measures. With emerging guidelines regarding patient-focused drug development from the FDA (16) and International Council of Harmonization and European Medicines Agency (17), there is a promising shift towards increased patient-centric research and regulatory processes.

## The therapeutic innovation journey and access to therapies to patients

It is important for all stakeholders involved in the therapeutic development lifecycle to understand the time, effort and the cost involved in bringing new therapies to market. Industry representatives highlighted the significant level resource required to bring a single drug to market: on average 2.5 billion USD (18), 14 years of development time, and the involvement of numerous stakeholders. Given the substantial investment and high risks involved in the research and development of novel therapies it is crucial for patients to maintain realistic expectations. This is especially true for rare eye diseases, where limited patient populations make it challenging for pharmaceutical companies to make a return on their investments. To maximize success and ensure treatments address outcomes that are meaningful to patients, pharmaceutical companies must prioritize patient involvement throughout the research and development process. Furthermore, there is usually a long delay in patients getting access to therapies even after regulatory approval. In Europe, Germany appears to have the highest rate of European Medicines Agency (EMA)-approved therapies (approved between 2018 and 2021) available (88%) to patients with Albania having the lowest number of EMA-approved therapies available (5%) (19). The average time between marketing authorization and availability to patients in Europe is 517 days. This is thought to be due to each Member State within the EU having its own Health Technology Assessment (HTA) body and distinct requirements.

In the European Union, there are steps being taken to streamline the HTA processes and to reduce this unacceptable waiting time. In the new proposed pathway, an EU-wide joint clinical assessment (EU JCA) platform will come into effect in January 2025 for oncology therapies, where companies will submit an EU JCA dossier, and the EU JCA outcome will be provided directly to individual countries. JCAs for orphan medicines are planned for 2028 (20).

The EU JCA will focus on assessing clinical evidence whereas the value judgment remains with the EU member states. With this new system the aim is to enhance efficiency in access to novel treatments for patients. Patients are experts of their own condition and can play a crucial role in educating HTA bodies and payers regarding the impact of their condition and, its impact and hence help improve access to therapies.

## Perspectives from patients, industry partners and academic clinicians on the value proposition of advanced therapeutic medicinal products (ATMPs) for inherited retinal degenerations and the role of patient voice

The panel discussion that included 3 patient representatives, 1 academic clinician and 1 chief medical officer from a pharmaceutical company primarily focused on the value proposition of advanced therapeutic medicinal products such as cell and gene therapies for Inherited Retinal Degenerations as well the importance of early and regular involvement of patients throughout the drug development journey. Several critical challenges and potential solutions in the development and accessibility of treatments were discussed.

The high cost and extended timeline of developing gene-specific therapies for over 300 genes associated with IRDs require the exploration of gene-independent therapies as well as alternative funding models. Panellists emphasized the importance of incorporating patient voices earlier in the drug development process, including in natural history studies and clinical trial designs. The discussion emphasized the difficulty in measuring and quantifying treatment value, particularly regarding quality-of-life improvements that is not captured by traditional clinical measures.

A Canadian panel member stated: “The first gene therapy for IRDs was approved for reimbursement in Canada in 2023, 6 years after approval in the USA, which is unacceptable. A 19-year-old male with low vision was treated within the last year. The patient mentioned that the biggest impact of this gene therapy treatment on his life was that he is discovering half of his life he did not know he had. This patient did not meet, criteria set by the regulators that determine efficacy, he did not have improvement in his visual acuity, and he actually lost some visual field. But his life has changed for the better. And not only his life changed, but his family’s life also changed because he’s better and he’s more independent. There are many parameters that we are not evaluating.” This is just an example how the traditional measures of vision are insufficient in determining a positive outcome in this particular patient population.

The need for better tools to assess patient-reported outcomes in IRD trials was stressed, along with the underestimation of social and economic costs of visual impairment in health technology assessments. Low rates of genetic testing among the affected population were also identified as a concern. The panel called for a new model of bringing therapies for rare diseases to patients, involving increased collaboration between industry, regulators, and patient groups. Panellists urged regulators to be more open-minded about alternative endpoints in clinical trials that demonstrate meaningful benefits to rare disease patients as opposed to focusing only on those generally accepted for more common diseases.

## Clinically meaningful vs. patient perceived benefit

The final discussion session was an opportunity for the audience members to interact with the presenters as well as the panel members to share their views on the topics discussed. Overall, there was agreement from the audience regarding the importance of the patient involvement throughout the lifecycle of therapeutic development, natural history studies and healthcare decision making such as regulatory and HTA bodies. Further, participants emphasized the importance of sharing both positive and negative results of clinical trials so that newer studies can build upon existing knowledge, avoid repeating unsuccessful approaches, and advance the field more efficiently.

There was a strong emphasis on incorporating the patient perspective on what is meaningful to patients into decision-making, with participants noting that stabilization of vision or slowing disease progression can be as valuable as improvement for many IRD patients. The discussion also touched on the misalignment between regulatory requirements for clinical benefit and what patients consider meaningful improvements in their quality of life. The importance of patient groups in shaping clinical trial designs and outcome measures was emphasized, as well as the need for better communication with both decision-makers and the general public about the realities of living with IRDs and the goals of treatment.

Overall, the conversation stressed the need for a more nuanced and patient-centered approach to assessing the value of specific IRD treatments.

## Conclusion

In conclusion, the aim of the RIWC2024 value introductory workshop and this perspective article is to improve literacy among patients, clinical experts, scientists and researchers as well as industry with regard to the unique and broad perspectives that patients can bring to research and decision-making processes, leading to better outcomes and more timely access to therapies for patients. All workshop attendees agreed that in the area of inherited retinal degenerations, where there is a slow progression of the disease over a long period of time, a collaborative effort between all stakeholders is critical in order to address the high unmet needs of this particular patient population. Recognizing the complexity of these conditions, stakeholders must embrace a specific and unique approach to evaluating the effects of innovative treatments. A follow-up workshop is planned at the RIWC26 conference in the United States in the year 2026, which will focus on further understanding of value-based care and role of patients in regulatory, health technology assessments and health-care decision making.

## Data Availability

The original contributions presented in the study are included in the article/supplementary material, further inquiries can be directed to the corresponding author.

## Workshop speakers and panellists

Avril Daly, Retina International, Ireland.

Russell Wheeler, LHON Society, UK,

Silvia Cerolini, Eyes on the Future, UK.

Stephan Huesler, Retina Suisse, Switzerland.

Tom Denee, PharmD, MBA, Johnson and Johnson Innovative Medicine.

Bart P Leroy, MD, PhD, Ghent University, Belgium.

Elise Heon, MD, FRCSC, SickKids Canada, Canada.

Marina Leite, Retina Brazil, Brazil.

Daniel Chung, DO, MA, Sparing Vision, France.

Sarah McLoughlin, PhD, Cancer Trials Ireland, Ireland.

Derick Mitchell, PhD, Irish Platform for Patients Organizations, Science and Industry (IPPOSI), Ireland.

Jason Menzo, Foundation Fighting Blindness, USA.
